# Global and tissue-specific transcriptomic dysregulation in human aging: Pathways and predictive biomarkers

**DOI:** 10.1007/s11357-025-01672-z

**Published:** 2025-04-28

**Authors:** Muhammad Arif, Andrea Lehoczki, György Haskó, Falk W. Lohoff, Zoltan Ungvari, Pal Pacher

**Affiliations:** 1 Laboratory of Cardiovascular Physiology and Tissue Injury, National Institute On Alcohol Abuse and Alcoholism, National Institutes of Health, Bethesda, MD USA; 2 Section On Fibrotic Disorders, National Institute and Alcohol Abuse and Alcoholism, National Institutes of Health, Bethesda, MD USA; 3 Department of Molecular and Clinical Medicine, SciLifeLab, Institute of Medicine, University of Gothenburg, Gothenburg, Sweden; 4 Doctoral College/Institute of Preventive Medicine and Public Health, International Training Program in Geroscience, Semmelweis University, Budapest, Hungary; 5 Department of Anesthesiology, Columbia University, New York, NY USA; 6 Section On Clinical Genomics and Experimental Therapeutics, National Institute On Alcohol Abuse and Alcoholism, National Institutes of Health, Bethesda, MD USA; 7 Vascular Cognitive Impairment, Neurodegeneration and Healthy Brain Aging Program, Department of Neurosurgery, University of Oklahoma Health Sciences Center, Oklahoma City, OK USA; 8 Oklahoma Center for Geroscience and Healthy Brain Aging, University of Oklahoma Health Sciences Center, Oklahoma City, OK USA; 9 Department of Health Promotion Sciences, College of Public Health, University of Oklahoma Health Sciences Center, Oklahoma City, OK USA; 10 The Peggy and Charles Stephenson Cancer Center, University of Oklahoma Health Sciences Center, Oklahoma City, OK USA

**Keywords:** Aging biomarkers, Transcriptomics, Gene co-expression networks, Cardiometabolic health, Mitochondrial dysfunction, Inflammation, Machine learning

## Abstract

Aging is a universal biological process that impacts all tissues, leading to functional decline and increased susceptibility to age-related diseases, particularly cardiometabolic disorders. While aging is characterized by hallmarks such as mitochondrial dysfunction, chronic inflammation, and dysregulated metabolism, the molecular mechanisms driving these processes remain incompletely understood, particularly in a tissue-specific context. To address this gap, we conducted a comprehensive transcriptomic analysis across 40 human tissues using data from the Genotype-Tissue Expression (GTEx) project, comparing individuals younger than 40 years with those older than 65 years. We identified over 17,000 differentially expressed genes (DEGs) across tissues, with distinct patterns of up- and down-regulation. Enrichment analyses revealed that up-regulated DEGs were associated with inflammation, immune responses, and apoptosis, while down-regulated DEGs were linked to mitochondrial function, oxidative phosphorylation, and metabolic processes. Using gene co-expression network (GCN) analyses, we identified 1,099 genes as dysregulated nodes (DNs) shared across tissues, reflecting global aging-associated transcriptional shifts. Integrating machine learning approaches, we pinpointed key aging biomarkers, including GDF15 and EDA2R, which demonstrated strong predictive power for aging and were particularly relevant in cardiometabolic tissues such as the heart, liver, skeletal muscle, and adipose tissue. These genes were also validated in plasma proteomics studies and exhibited significant correlations with clinical cardiometabolic health indicators. This study provides a multi-tissue, integrative perspective on aging, uncovering both systemic and tissue-specific molecular signatures. Our findings advance understanding of the molecular underpinnings of aging and identify novel biomarkers that may serve as therapeutic targets for promoting healthy aging and mitigating age-related diseases.

**Supplementary Information:**

The online version contains supplementary material available at 10.1007/s11357-025-01672-z.

## Introduction

Aging is a universal biological process that impacts all tissues, leading to a progressive decline in physiological functions and an increased susceptibility to age-related diseases, particularly cardiometabolic disorders. While hallmarks of aging, such as genomic instability, mitochondrial dysfunction, chronic inflammation, and dysregulated energy metabolism, have been identified, their manifestation varies significantly across tissues, reflecting the complexity and heterogeneity of aging [1]. Understanding these tissue-specific and systemic changes is essential for unraveling the molecular mechanisms driving aging.

Despite advancements in aging research, significant gaps remain in our understanding of how these hallmarks interplay across different tissues in humans. Most studies have focused on single tissues or small datasets, limiting the ability to derive comprehensive insights into the molecular signatures that underpin aging at a multi-tissue or systemic level. Additionally, while research in model organisms has provided critical insights into conserved aging pathways—such as those involving nutrient sensing, mitochondrial function, and oxidative stress—translation of these findings to human biology remains incomplete. Existing studies often confirm known pathways without exploring novel patterns or integrating data at a global scale, leaving potential tissue-specific or pan-tissue biomarkers unexplored.

Recent advancements in transcriptomics have enabled the large-scale profiling of gene expression changes associated with aging, offering the opportunity to identify tissue-specific and systemic biomarkers [2–11]. However, a critical gap persists in the integration of these datasets across tissues to uncover aging’s broader molecular landscape. For instance, while it is well-established that metabolic and inflammatory pathways are dysregulated in aging, the extent to which these processes are shared or distinct across tissues remains unclear. Moreover, how these processes contribute to age-related diseases such as cardiovascular and metabolic disorders at the transcriptomic level is still poorly understood.

Leveraging large-scale datasets such as those from the Genotype-Tissue Expression (GTEx) project allows for a more integrative and comprehensive investigation of the aging process [3]. By comparing transcriptomic profiles across 40 human tissues, this study aims to address key questions: What are the shared and tissue-specific transcriptional signatures of aging? Which genes and pathways consistently drive the aging process across tissues, and how are these linked to age-related cardiometabolic diseases?

We hypothesize that aging drives both global (shared) and localized (tissue-specific) transcriptional dysregulation, reflecting systemic processes such as mitochondrial decline and inflammation, alongside tissue-specific vulnerabilities. To investigate this, we performed differential gene expression analyses across 40 human tissues, complemented by gene co-expression network (GCN) analyses to identify dysregulated nodes (DNs) that characterize changes in aging-associated networks. Using machine learning approaches, we prioritized key biomarkers with strong predictive power for aging, including *GDF15* and *EDA2R*, which emerged as particularly relevant in cardiometabolic tissues. Our study aims to unravel the complex molecular landscape of aging by identifying both systemic and tissue-specific transcriptional changes. We seek to characterize the molecular pathways most affected by aging across tissues and to pinpoint robust biomarkers that have potential relevance to cardiometabolic health. By integrating transcriptomic data with advanced analytical approaches, this work provides a comprehensive, multi-tissue perspective on aging. Ultimately, our findings advance the understanding of aging’s molecular underpinnings, offering new insights into pathways and biomarkers that may serve as targets for promoting healthy aging and mitigating age-related diseases.

## Material and methods

### Human data source and selections

In this study, we used transcriptomics data from The Adult Genotype Tissue Expression (GTEx) Project on 02/08/2022. The human transcriptomics data, including raw counts and TPM values, were downloaded from the GTEx Portal, specifically from the “GTEx Analysis V8” section. Extended phenotype data was retrieved from dbGAP accession number phs000424.v8.p2. We stratified the subjects based on Young (< 40 years old) and Aging (> 65 years old) groups. The data was further filtered by removing the known diseases and causes of death associated with the tissues. We analyzed only the tissues that had more than 10 samples per group. The sample size and exclusion criteria details can be found in the Supplementary Table 1.

### Differential expression analysis

The differential expression analysis for each tissue was done using a previously described pipeline [12]. In short, the raw count data from each tissue was used as the input for DESeq2 [13], and genes with Adjusted P-Values < 0.05 were considered significant. Batch information was included in the model matrix of DESeq2. Directions of the changes were defined by their Log2 Fold Change with no specific cut-off. The differentially expressed genes (DEGs) were categorized into categories described by the Human Protein Atlas [14]. The categorization of “DEGs in Single”, “DEGs in Some”, and “DEGs in Many” is discussed further in the appropriate results sections.

### Co-expression network analysis

Co-expression network analysis for each tissue and age group was performed using a previously described by iNetModels [15]. In short, we performed Spearman correlation analysis on the TPM values using the *spearmanr* function from SciPy 1.7.3 in Python 3.7. Only genes with TPM values > 5 and the gene–gene correlation with FDR < 0.05 were used for further centrality analysis (degree) using iGraph 0.9.11. The dysregulated network analysis focused on the difference in degree centrality in the positively correlated genes. The same categorization rationale as the DEGs was employed. The network from the SCAPIS-SciLifeLab longitudinal study was downloaded directly from https://inetmodels.com [15] and visualized by Cytoscape.

### Random forest analysis

We employed a random forest classifier function from the Scikit Learn 1.0.2 package in Python 3.7. The data was split into train and test groups with a proportion of 70% and 30%, respectively, for overall and tissue-specific classification. We enabled the bootstrap and out-of-bag estimation with 1000 trees. ROC curve was generated using the *RocCurveDisplay* function.

### AllOfUs data analysis

We use the AllOfUs cohorts (selection criteria described in the results section) into Young (< 40 years old), Middle (40–65), and Aging (> 65) groups. We used PhecodeX [16] to map the diseases to organ groups based on their International Classification of Diseases codes. We removed all congenital and genetic diseases from the data. To identify the age-related diseases, we employed the Binomial Generalized Linear Model using the *glm* function from Statsmodels in Python. We considered alcohol, marijuana, smoking, race, and gender as confounding factors and included them in the model. Only diseases with FDR < 0.05 is considered as significantly associated with aging process.

### Functional gene analysis

Functional analysis of the up-and down-regulated genes was performed using the GSEAPY package in Python 3.7 with gene-set collections from the Enrichr [17, 18] website.

## Results

### Study design and sample distribution

**Fig. 1:**
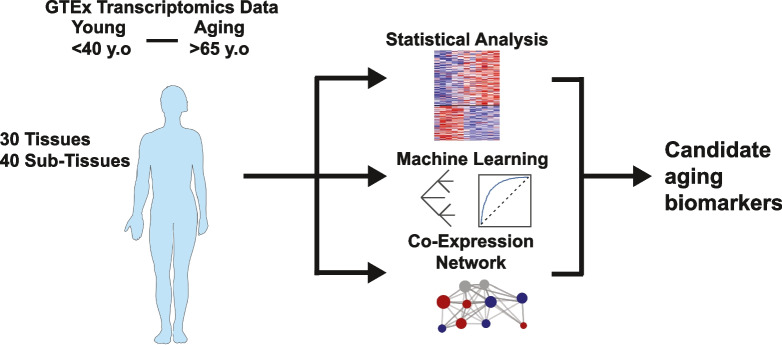
Study Overview and Analysis Summary

The study utilized transcriptomic data from the Genotype-Tissue Expression (GTEx) project, encompassing 40 human tissues from individuals classified into two age groups: young (< 40 years) and aging (> 65 years) (Fig. 1). This dataset provides a comprehensive and standardized resource for investigating aging-associated molecular changes across tissues. The total number of samples analyzed was designed to capture both tissue-specific and systemic aging signatures while minimizing confounding factors such as underlying diseases. Key tissues relevant to cardiometabolic health, including the heart, liver, skeletal muscle, and adipose tissue, were given particular focus. Details of the analyzed tissues and the number of subjects can be found in the Supplementary Table 1.

### Classification of differentially expressed genes (DEGs) reveals tissue-specific and systemic aging responses

**Fig. 2:**
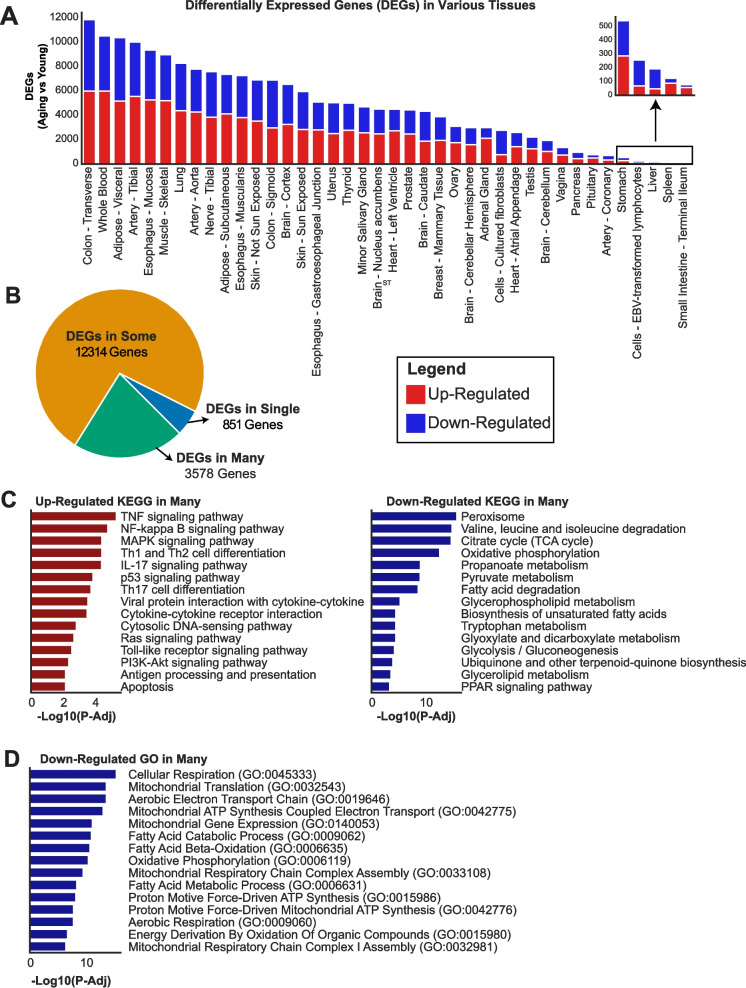
Differential Expression Analysis of Young (< 40 years old) and Aging (> 65 years old) groups across tissues. (**A**) Bar chart depicting the number of differentially expressed genes (DEGs) across 40 human tissues. Up-regulated genes are shown in red, and down-regulated genes in blue. Tissues with the highest number of DEGs include the transverse colon, whole blood, and adipose tissue, while the terminal ileum and spleen exhibit the fewest transcriptional changes. The inset zooms in on tissues with fewer than 600 DEGs (**B**) Pie chart categorizing DEGs based on their tissue distribution."DEGs in Single"(851 genes) are restricted to individual tissues,"DEGs in Some"(12,314 genes) are shared between 2 to 13 tissues, and"DEGs in Many"(3,578 genes) are observed across more than 14 tissues. The classification is based on the detection classification of the Human Protein Atlas. (**C**) KEGG pathway enrichment analysis for DEGs observed in many tissues. Up-regulated pathways (left) include inflammation and immune signaling (e.g., TNF signaling, NF-kappa B pathway, apoptosis). Down-regulated pathways (right) include metabolic and mitochondrial processes (e.g., TCA cycle, oxidative phosphorylation, fatty acid metabolism). (**D**) Gene Ontology (GO) enrichment analysis for down-regulated DEGs observed in many tissues, showing a significant decline in mitochondrial and energy generation processes, including oxidative phosphorylation, ATP synthesis, and fatty acid degradation. This figure demonstrates the tissue-specific and systemic transcriptional changes associated with aging, highlighting the increased inflammatory response and metabolic decline as hallmarks of the aging process

The transcriptomic analysis revealed a wide variability in the number of differentially expressed genes (DEGs) across tissues, reflecting distinct aging-related transcriptional remodeling. The number of DEGs ranged from as few as 134 in the terminal ileum to as many as 17,873 in the transverse colon, the tissue with the highest DEG count (Fig. 2A, Supplementary Table 1, Supplementary Table 2). Other tissues with substantial transcriptional remodeling included whole blood, visceral and subcutaneous adipose tissue, and tibial artery, indicative of their metabolic or immunological activity. Conversely, tissues like the spleen and liver exhibited relatively modest transcriptional changes, suggesting lower sensitivity or resistance to aging-related dysregulation. To better understand the distribution of DEGs across tissues, we categorized them into three groups [14]:

1. DEGs observed in single tissues (5.1%; 851 genes): These were unique to individual tissues, highlighting tissue-specific aging responses. Notable examples include whole blood (89 DEGs), brain cortex (81 DEGs), and testis (68 DEGs) (Fig. 2B, Supplementary Table 1).
2. DEGs observed in some tissues (73.5%; 12,314 genes): These were shared across 2–13 tissues, often among those with similar developmental or functional origins, such as the transverse and sigmoid colon or different brain regions.
3. DEGs observed in many tissues (21.4%; 3,578 genes): These genes were observed in more than 14 tissues, representing systemic aging responses and potentially universal aging markers.

#### Functional insights into DEGs observed in many tissues

Functional enrichment analysis of DEGs observed in many tissues revealed hallmark aging processes. Up-regulated DEGs were predominantly associated with inflammation, immune responses, and apoptosis, including pathways such as TNF signaling, NF-kappa B signaling, and MAPK signaling (Fig. 2C). These findings align with the well-documented pro-inflammatory state observed during aging.

Conversely, down-regulated DEGs were enriched in pathways reflecting metabolic decline, including oxidative phosphorylation, fatty acid metabolism, and the TCA cycle (Fig. 2C). Gene Ontology (GO) enrichment analysis revealed that down-regulated DEGs were significantly associated with mitochondrial functions and energy generation processes, including ATP synthesis, electron transport chain activity, and fatty acid metabolism (Fig. 2D). These findings underscore the central role of mitochondrial dysfunction in aging, with reduced energy production emerging as a hallmark across multiple tissues. The suppression of these critical processes aligns with the metabolic decline observed in aging individuals and highlights potential targets for interventions aimed at mitigating age-related functional deterioration. This dual pattern of increased inflammation and diminished metabolic function underscores the molecular complexity of aging.

#### Tissue-specific aging responses

Zooming into tissue-specific DEGs, certain patterns emerged. Tissues such as whole blood, brain cortex, and testis showed a high proportion of unique DEGs, reflecting their specialized roles and aging dynamics. The “DEGs in Some” category included genes shared among tissues with similar embryological origins, such as different skin types or colon segments. This suggests that aging processes are partially conserved within related tissues.

Overall, the classification and functional analysis of DEGs highlights both tissue-specific and systemic aspects of aging. While inflammation and immune responses are universally up-regulated across tissues, metabolic processes such as mitochondrial function and oxidative phosphorylation are consistently down-regulated. These results provide a foundation for identifying both global biomarkers and tissue-specific therapeutic targets for aging (Fig. 2B-D).

### Dysregulated gene co-expression networks reveal key drivers of aging

**Fig. 3:**
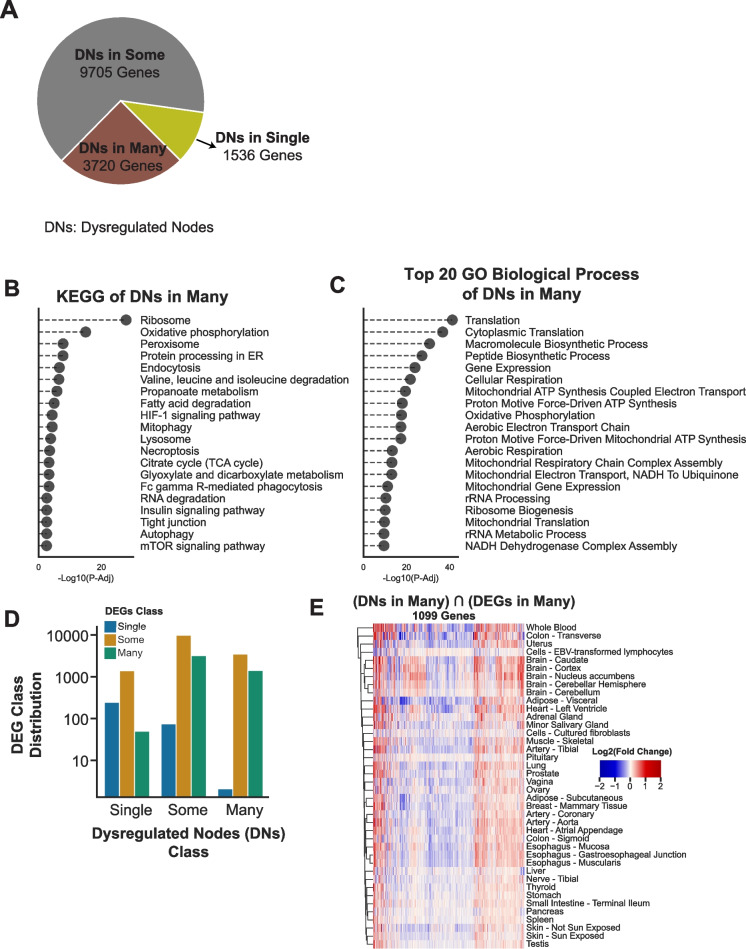
Dysregulated Gene Co-expression Networks (DNs) in Aging. (**A**) Pie chart categorizing DNs based on their tissue distribution."DNs in Single"(1,536 genes) are restricted to individual tissues,"DNs in Some"(9,705 genes) are shared across 2–13 tissues, and"DNs in Many"(3,720 genes) are common to more than 14 tissues, representing systemic aging signatures. (**B**) KEGG pathway enrichment analysis for DNs in Many tissues. Up-regulated pathways include oxidative phosphorylation, ribosome biogenesis, and peroxisome function. Other pathways impacted by aging include fatty acid metabolism, lysosome activity, mitophagy, necroptosis, and HIF- 1 signaling. (**C**) Top 20 GO Biological Processes associated with DNs in Many tissues. Processes such as translation, mitochondrial ATP synthesis, cellular respiration, and ribosomal RNA metabolic processes dominate, highlighting mitochondrial and translational dysregulation in aging. (**D**) Comparison of DEG and DN classifications. The bar graph shows the distribution of genes classified as"Single,""Some,"or"Many"for both DEGs and DNs, emphasizing the complementary insights provided by each analysis method. (**E**) Heatmap showing the Log2 Fold Change (L2 FC) of 1,099 genes identified as both DNs in Many and DEGs in Many. Genes exhibit consistent patterns of dysregulation across multiple tissues, with clustering by tissue type revealing shared aging-related transcriptional changes

To further explore molecular changes associated with aging, we conducted a dysregulated network (DN) analysis to identify genes with significant shifts in co-expression network centrality between young (< 40 years) and aging (> 65 years) groups. These Dysregulated Nodes (DNs) capture altered connectivity within gene co-expression networks, reflecting their potential role in driving phenotypic differences during aging. We selected top 10% of the most dysregulated nodes, based on the changes in their degree connectivity in aging and young co-expression networks. DNs were classified based on their tissue distribution:

1. DNs in single tissues (1,536 genes): Restricted to individual tissues, these nodes highlight tissue-specific aging changes. The testis showed the highest number of single-tissue DNs, followed by whole blood and the cerebellum.
2. DNs in some tissues (9,705 genes): Shared across 2–13 tissues, these nodes often involve tissues with related functions or developmental origins.
3. DNs in many Tissues (3,720 genes): Observed in more than 14 tissues, these nodes represent systemic aging responses (Fig. 3A).

Functional enrichment analysis revealed that DNs identified in many tissues were closely linked to hallmark pathways of aging. Among these, mitochondrial and metabolic processes stood out, encompassing pathways such as oxidative phosphorylation, fatty acid degradation, the TCA cycle, and ATP synthesis. These findings point to widespread mitochondrial dysfunction, a central feature of aging (Fig. 3B). In addition to metabolic decline, cellular stress responses were evident through the enrichment of pathways involved in ribosome biogenesis, lysosome function, autophagy, and mitophagy, highlighting the cellular mechanisms engaged in maintaining homeostasis under aging-related stress.

Gene Ontology (GO) enrichment analysis further supported these observations, emphasizing processes related to translation and RNA metabolic activities. These findings align with the broader decline in energy production and protein synthesis that characterizes aging (Fig. 3C). A comparative analysis of DEGs and DNs revealed complementary insights into aging biology. DEGs focus on changes in gene expression levels, identifying genes directly up- or down-regulated across tissues during aging. DNs emphasize changes in network connectivity, offering a deeper understanding of the dynamic interactions and structural shifts within gene co-expression networks during aging. Together, these analyses provide a comprehensive view of the transcriptional and network-level changes associated with the aging process. DN analysis identified a larger proportion of tissue-specific nodes compared to DEG analysis, demonstrating its strength in capturing network dynamics alongside transcriptional changes. This distinction highlights the value of integrating these approaches to achieve a more comprehensive understanding of aging processes (Fig. 3D).

To refine global aging biomarkers, we integrated DEG and DN findings, narrowing the focus to 1,099 genes classified as both “DEGs in Many” and “DNs in Many” (Fig. 3E). These genes exhibited consistent patterns of up- or down-regulation across multiple tissues. Clustering by tissue type further revealed shared transcriptional trends across aging-related processes. Enrichment analysis of these 1,099 genes identified strong associations with metabolic pathways, including fatty acid, lipid, and carbohydrate metabolism, as well as cellular processes like apoptosis and focal adhesion. These findings underscore the systemic impact of these pathways on aging and their relevance as global biomarkers.

### Machine learning analysis identifies top genes to predict the aging process

**Fig. 4:**
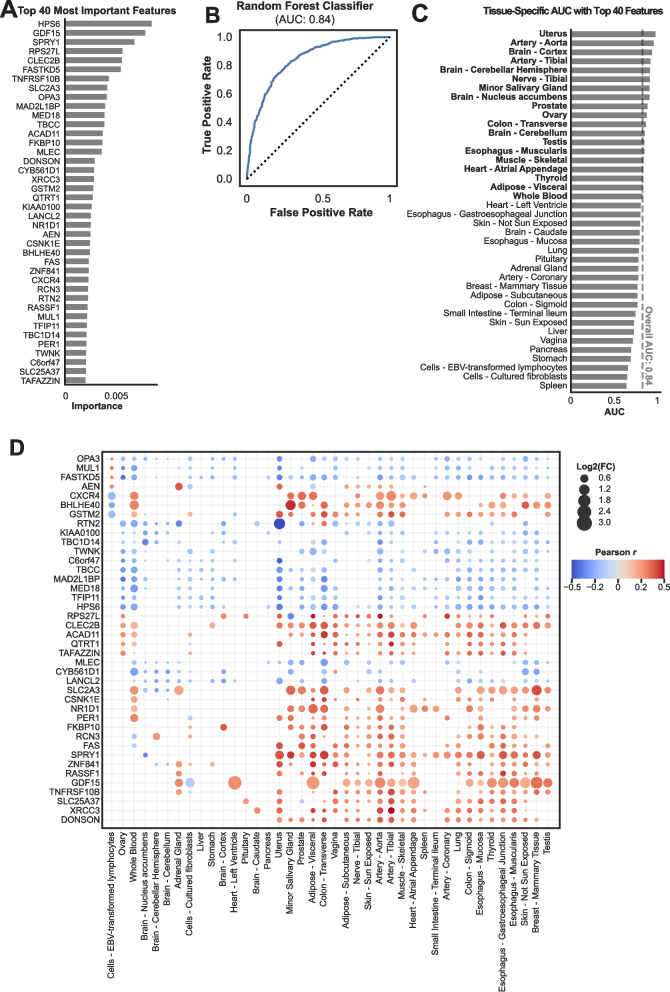
Random Forest Analysis Identifying Key Genes and Tissue-Specific Aging Signatures. (**A**) Bar chart showing the top 40 most important features (genes) identified by the random forest classifier in predicting aging across all tissues. Genes such as *HPS6*, *GDF15*, *SPRY1*, and *RPS27L* show the highest importance, underscoring their relevance as systemic aging markers. (**B**) Receiver Operating Characteristic (ROC) curve for the random forest model trained on the top 40 features, with an Area Under the Curve (AUC) of 0.84, indicating robust classification performance in distinguishing between young (< 40 years) and aging (> 65 years) groups. (**C**) Tissue-specific AUC values for the random forest model using the top 40 features across different tissues. Tissues such as the uterus, testis, cortex, and aorta show the highest performance (AUC > 0.84), suggesting strong predictive capacity for aging in these tissues. Conversely, tissues like the spleen and cultured fibroblasts have lower predictive performance. (**D**) Bubble plot showing the Log2 Fold Change (L2 FC) and Pearson correlation of the top 40 genes with age across tissues. Bubble size reflects the magnitude of fold change, while color indicates the direction and strength of correlation. Genes such as *GDF15*, *SPRY1*, and *CLEC2B* show consistent positive correlations with age across multiple tissues, while others (e.g., *HPS6*) exhibit strong negative correlations, particularly in metabolic and mitochondrial tissues

To identify key genes predictive of aging across tissues, we applied a random forest machine learning approach, focusing on the 1,099 genes classified as both “DEGs in Many” and “DNs in Many.” This analysis highlighted 40 top-ranking genes with significant predictive power in distinguishing young (< 40 years) from aging (> 65 years) individuals (Fig. 4A).

Among the top 40 genes identified, HPS6, GDF15, SPRY1, and RPS27L emerged as the most critical predictors of aging, reflecting their roles in mitochondrial function, inflammation, and apoptosis—processes central to aging biology. Pathway enrichment analysis revealed strong associations with circadian rhythm (e.g., *NR1D1*, *CSNK1E*, *BHLHE40*, *PER1*), cytokine-cytokine receptor interactions (e.g., *GDF15*, *TNFRSF10B*, *CXCR4*, *FAS*), and p53 signaling pathways (e.g., *FAS*, *TNFRSF10B*). Other enriched processes included mitochondrial RNA metabolism, underscoring the relevance of these genes in aging-associated pathways.

The random forest model achieved robust predictive performance, with an Area Under the Curve (AUC) of 0.84, an accuracy of 76%, and an out-of-bag (OOB) score of 75% (Fig. 4B). This performance underscores the effectiveness of the identified genes as aging biomarkers.

#### Tissue-specific performance

Using the top 40 genes, we evaluated the predictive performance in individual tissues. Nineteen tissues, including sex-specific organs (uterus, testis, ovary, and prostate), the aorta, tibial artery, brain regions (except caudate), transverse colon, tibial nerve, esophagus, atrial appendage, skeletal muscle, whole blood, thyroid, and visceral adipose tissue, achieved an AUC greater than 0.84, outperforming the overall model (Fig. 4C; Supplementary Fig. 1). Conversely, tissues such as the spleen and cultured fibroblasts exhibited lower predictive performance, reflecting variability in aging-related transcriptomic changes across tissues.

#### Correlation of key genes with aging

Correlation analysis of the top 40 genes with age revealed that 28 genes were positively correlated with age, while 12 showed negative correlations in the majority of tissues (Fig. 4D). Several notable trends were observed. *GDF15*, *SPRY1*, *RPS27L*, and *CLEC2B* were consistently positively correlated with age across 18–25 tissues, including the aorta, tibial artery, lung, skeletal muscle, subcutaneous adipose, tibial nerve, esophagus, and reproductive organs. HPS6, the top-ranked gene, showed a strong negative correlation with age in 30 tissues, particularly those with high metabolic activity (e.g., liver, skeletal muscle, and adipose tissue). Genes involved in apoptosis and inflammation, such as *FAS*, *CXCR4*, and *TNFRSF10B*, demonstrated positive correlations with age across most tissues, except for *FAS* in whole blood. Mitochondrial-associated genes, including *TWNK* and *FASTKD5*, were negatively correlated with age, reflecting mitochondrial decline during aging.

This analysis highlights apoptosis, mitochondrial processes, and fatty acid metabolism as critical pathways underlying aging. Genes such as *GDF15* and *HPS6* stand out as strong candidates for systemic aging biomarkers due to their consistent correlations across tissues. These findings demonstrate the utility of machine learning approaches in identifying tissue-specific and systemic biomarkers, offering a valuable foundation for further validation and exploration of potential anti-aging therapeutic targets (Fig. 4A-D).

### AllofUs data highlights the prevalence of cardiovascular, metabolic, and musculoskeletal disease in aging populations

To further validate our framework for identifying whole-body aging signatures, we applied it to a specific subsystem of the body using population-based data from the NIH AllofUs study. This extensive dataset provided a unique opportunity to examine the relationship between aging and disease prevalence across a diverse cohort.

We analyzed data from 22,279 participants aged 20 to 80 years, with BMIs between 18.5 and 30, who met strict inclusion criteria to minimize confounding factors. Participants were selected based on comprehensive electronic health records and detailed information on alcohol and marijuana consumption. Individuals with conditions such as pregnancy, wheelchair use, or a history of substance abuse were excluded from the study. This careful selection ensured a robust dataset for evaluating the impact of aging on disease risk.

The analysis identified 291 conditions significantly associated with aging. Among these, more than 40% were linked to the decline of cardiometabolic systems, encompassing cardiovascular, metabolic, and musculoskeletal diseases. Cardiovascular diseases, such as hypertension, atherosclerosis, and heart failure, emerged as major contributors to the aging burden. Metabolic disorders, including type 2 diabetes and dyslipidemia, highlighted the systemic metabolic dysregulation associated with aging. Additionally, musculoskeletal diseases, such as osteoarthritis and sarcopenia, reflected age-related deterioration in muscle and bone health. These findings underscore the disproportionate impact of aging on these critical systems (Supplementary Fig. 2). This analysis highlights the utility of our integrative approach in linking molecular aging signatures to clinical outcomes observed in real-world populations. The high prevalence of cardiometabolic and musculoskeletal diseases emphasizes the need to prioritize these subsystems in aging research. By integrating transcriptomic data with clinical insights, we can deepen our understanding of the molecular mechanisms underlying these age-associated conditions and pave the way for targeted prevention and intervention strategies.

### Analysis of heart, liver, adipose, and skeletal muscle reveals tissue- and subsystem-specific aging markers

Focusing on four key tissues associated with the cardiometabolic system—heart (left ventricle and atrial appendage), liver, adipose (subcutaneous and visceral), and skeletal muscle—we identified tissue-specific aging markers by selecting genes that were both differentially expressed and dysregulated in gene co-expression networks. Random forest analysis was then applied to pinpoint the most influential genes in each tissue.

**Fig. 5:**
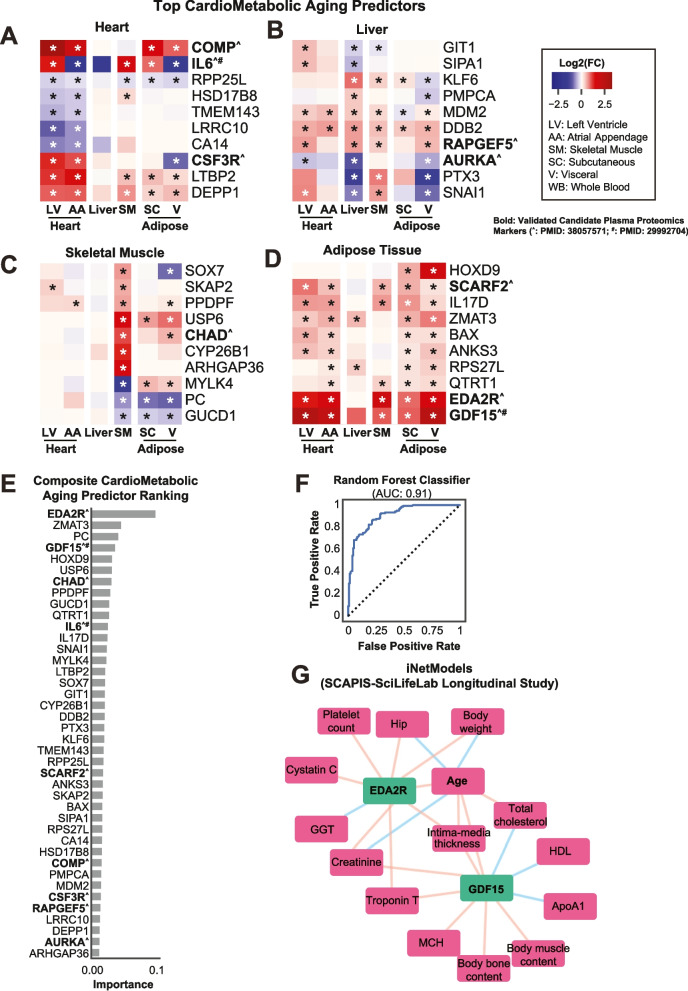
Tissue-Specific and Composite Cardiometabolic Aging Biomarkers. (**A**-**D**) Heatmaps of top aging predictors identified in cardiometabolic tissues (heart, liver, skeletal muscle, and adipose tissue). Genes are ranked by their Log2 Fold Change (L2 FC) values in left ventricle (LV), atrial appendage (AA), liver, skeletal muscle (SM), and adipose tissue (subcutaneous [SC] and visceral [V]). Validated plasma biomarkers are indicated in bold, with genes validated in candidate plasma proteomics studies denoted by an asterisk (*). Notable markers include *COMP, IL6, SCARF2, EDA2R*, and *GDF15*, which exhibit consistent changes across multiple tissues. (**E**) Composite cardiometabolic aging predictor ranking derived from a random forest model using features from panels A-D. *EDA2R* and *GDF15* emerged as the top predictors, demonstrating their importance in both systemic and tissue-specific aging. (**F**) Receiver Operating Characteristic (ROC) curve showing the predictive performance of the composite model (AUC: 0.91). The model achieved high accuracy, validating the utility of these predictors in identifying aging-related transcriptomic patterns. (**G**) Network analysis of *EDA2R* and *GDF15* in the SCAPIS-SciLifeLab longitudinal study. Positive correlations (green lines) and negative correlations (red lines) with various clinical variables (e.g., total cholesterol, high-density lipoprotein [HDL], and body muscle content) highlight their association with cardiometabolic and systemic health during aging

In the heart, the top 10 genes identified by the model (accuracy: 0.80, out-of-bag [OOB] score: 0.72) included markers of fibrosis and inflammation, such as *COMP*, *IL6*, and *CSF36*. These genes are closely linked to structural remodeling and immune activity during aging. Additionally, *HSD17B8* (fatty acid metabolism) and *RPP25L* (ribosome biogenesis) highlighted the role of metabolic and translational processes in cardiac aging. Other key genes included *TMEM143, LRRC10, CA14, LTBP2*, and *DEPP1*, further reflecting the complexity of transcriptomic changes in aging cardiac tissue (Fig. 5A).

The top liver genes (accuracy: 0.90, OOB score: 0.70) revealed pathways associated with cellular senescence and mitochondrial processing. Genes such as *MDM2* and *DDB2*, involved in p53 signaling and DNA damage repair, and AURKA, known for its role in senescence, were among the top markers. Mitochondrial protein processing was represented by *PMPCA*, while Ras signaling was reflected in *RAPGEF5* and *SIPA1*. Inflammatory responses were captured by *PTX3*, and cytokinesis regulation by *GIT1*, illustrating the interplay of metabolic, stress response, and regulatory pathways in hepatic aging (Fig. 5B).

In skeletal muscle (accuracy: 0.84, OOB score: 0.78), aging markers included *CHAD* and *MYLK4*, linked to focal adhesion and muscle structure. Metabolic decline was indicated by PC (pyruvate metabolism), while bone morphogenesis and retinoic acid signaling were captured by *CYP26B1*. Other notable genes, such as *SOX7* (apoptosis and Wnt signaling) and *USP6* (protein deubiquitination), highlighted cellular remodeling processes. Genes like *SKAP2, PPDPF, ARHGAP36*, and *GUCD1* further underlined the diversity of pathways affected in muscle aging (Fig. 5C).

In adipose tissue (accuracy: 0.76, OOB score: 0.76), key markers included *GDF15*, *IL17D*, and *EDA2R*, which are involved in cytokine-cytokine receptor interactions and inflammatory signaling. Markers of apoptotic processes, such as *BAX* and *RPS27L*, as well as *ZMAT3* (p53 signaling), reflect stress-induced responses in adipose aging. Other significant genes included *SCARF2* (LDL degradation), *HOXD9*, *ANKS3*, and *QTRT1*, underscoring metabolic and regulatory shifts associated with aging adipose tissue (Fig. 5D).

These analyses reveal distinct, tissue-specific transcriptomic changes in the cardiometabolic system, highlighting both shared and unique molecular mechanisms underlying aging. The identification of key markers such as *COMP* in the heart, *MDM2* in the liver, *SOX7* in skeletal muscle, and *GDF15* in adipose tissue emphasizes the interplay of inflammation, metabolism, and structural remodeling in tissue-specific aging processes. These findings pave the way for targeted interventions to mitigate the impacts of aging on these critical tissues.

### Composite analysis of cardiometabolic aging predictors reveals EDA2R and GDF15 as candidate biomarkers and therapeutic targets

Although the aging-associated genes identified in each cardiometabolic tissue were unique, their overall trends of up- and down-regulation were consistent across all four tissues (Fig. 5A-D). This observation led us to hypothesize that combining these genes could enhance the identification of composite predictors for cardiometabolic tissue aging. To test this, we integrated the expression profiles of the 40 top genes from the individual tissue analyses and used them as input for a random forest analysis encompassing all four tissues (Fig. 5E-F).

The composite model demonstrated strong predictive performance, achieving an AUC of 0.91, an accuracy of 0.8, and an out-of-bag (OOB) score of 0.78. From this analysis, *EDA2R*, *ZMAT3*, *PC*, *GDF15*, and *HOXD9* emerged as the top five genes based on feature importance. Notably, *EDA2R* and *GDF15* were consistently up-regulated across all cardiometabolic tissues (Fig. 5D) and have previously been validated as elevated in aging plasma proteomics studies, underscoring their potential as systemic aging biomarkers.

To further explore their relevance, we examined data from the SCAPIS-SciLifeLab wellness profiling longitudinal study using iNetModels (Fig. 5G). Both *EDA2R* and *GDF15* demonstrated positive correlations with age. *EDA2R* was associated with increased levels of cystatin C, platelet count, body weight, and hip measurement, as well as reduced levels of gamma-glutamyl transferase (GGT), a liver enzyme. Similarly, *GDF15* showed positive correlations with mean corpuscular hemoglobin (MCH), body bone, and muscle content, while exhibiting negative correlations with high-density lipoprotein (HDL), total cholesterol, and Apolipoprotein A1 (ApoA1). These findings suggest a robust association between these biomarkers and metabolic and musculoskeletal system health. Interestingly, both *EDA2R* and *GDF15* also demonstrated positive correlations with cardiovascular health indicators, including Troponin T and intima-media thickness, as well as creatinine levels, further reinforcing their relevance to cardiometabolic aging.

Taken together, the composite analysis highlights *EDA2R* and *GDF15* as promising candidate biomarkers and therapeutic targets for cardiometabolic aging. Their consistent up-regulation across tissues and strong correlations with clinical metrics related to metabolic, musculoskeletal, and cardiovascular health underscore their systemic significance in the aging process. These findings offer a foundation for future studies aimed at validating their utility in aging diagnostics and therapeutic interventions.

## Discussion

Aging is an inevitable biological process that impacts all tissues and is a major risk factor for complex diseases. This study systematically explored the molecular underpinnings of aging across the human body by integrating transcriptomic data from 40 tissues with advanced statistical, network, and machine-learning approaches. By stratifying subjects into young (< 40 years) and aging (> 65 years) groups and removing individuals with tissue-related diseases or significant underlying conditions, we captured a clearer picture of transcriptional changes associated with healthy aging. This comprehensive analysis provides new insights into systemic and tissue-specific markers of aging, while emphasizing the importance of cardiometabolic tissues as key contributors to age-related health decline.

Our analysis revealed significant transcriptional changes across all 40 tissues, with more than 3,500 genes differentially expressed in over 30% of the tissues. These commonly dysregulated genes were enriched in hallmark pathways of aging [1], including mitochondrial dysfunction, reduced energy production, and down-regulated metabolic pathways such as oxidative phosphorylation, glycolysis, and fatty acid metabolism. The observed decline in mitochondrial and energy-related processes aligns with the well-established notion that metabolic efficiency diminishes with age, contributing to systemic dysfunction [19–21]. Concurrently, up-regulated genes were associated with inflammation and immune responses, including TNF, NF-kappa B, and IL- 17 signaling pathways, as well as apoptotic processes via p53 signaling. These findings underscore a dual pattern of metabolic decline and heightened inflammatory states as central features of aging [20, 22, 23].

We introduced a novel DN approach, which evaluates changes in gene connectivity between young and aging co-expression networks. This method identified 3,720"DNs in Many"genes that were associated with aging across tissues. These nodes captured a larger proportion of common signatures than conventional DGE analysis, while also doubling the number of tissue-specific aging genes. This improved sensitivity of DN analysis highlights its potential as a robust alternative to conventional methods, particularly in identifying key drivers of tissue-specific and systemic aging processes.

The DN approach also validated its utility by identifying externally validated tissue-specific aging signatures missed by traditional DGE methods. Notably, DN-derived genes were strongly associated with hallmark aging pathways, including mitochondrial processes, translation, and autophagy, reinforcing their relevance to the aging phenotype.

### Machine learning predictors of aging

Using machine learning, we ranked protein-coding genes based on their importance in predicting whole-body aging, achieving an AUC of 0.84. Among the top predictors were *GDF15*, *HPS6*, and *EDA2R*, which demonstrated strong tissue-specific and systemic relevance. *GDF15*, in particular, emerged as a key whole-body biomarker, consistently associated with mitochondrial dysfunction and inflammatory processes. Similarly, EDA2R, a member of the TNF receptor family, was highlighted as a significant marker for cardiometabolic aging due to its association with obesity, insulin resistance, and muscle atrophy.

### Cardiometabolic aging and tissue-specific markers

The focus on cardiometabolic tissues—heart, liver, skeletal muscle, and adipose—revealed distinct yet interconnected aging signatures. These tissues displayed transcriptional changes associated with fibrosis, inflammation, and metabolic decline, underscoring their central role in cardiometabolic health. Notably, the composite analysis of aging predictors identified *EDA2R* and *GDF15* as systemic markers strongly correlated with clinical metrics of cardiometabolic health, including total cholesterol, HDL, cardiac troponin, and gamma-glutamyltransferase (GGT). These robust associations reinforce the relevance of *EDA2R* and *GDF15* as biomarkers and potential therapeutic targets for mitigating cardiometabolic aging.

### GDF15 as a biomarker in aging

*GDF15* (also known as Macrophage inhibitory cytokine- 1 (MIC- 1)) has emerged as a prominent marker of aging in both experimental and clinical studies. A member of the TGF-β superfamily, *GDF15* is upregulated in response to cellular stress, including mitochondrial dysfunction [24], oxidative stress, and inflammation, all of which are hallmark processes of aging. Its role as a circulating biomarker of aging has been extensively validated across model organisms and human studies, making it a focus of recent aging research. Mitochondrial stress, a critical driver of aging, has been shown to upregulate *GDF15* [24]. These findings reinforce its role as a systemic responder to mitochondrial and metabolic stress, linking it to the broader decline in cardiometabolic function observed during aging. *GDF15* has been closely linked to cellular senescence, a hallmark of aging characterized by irreversible cell cycle arrest and secretion of pro-inflammatory factors [25–29]. *GDF15* is secreted by senescent cells, where it functions as part of the senescence-associated secretory phenotype (SASP) [27, 28]. With aging senescent cell burden increases across various tissues, which contributes to the elevation of circulating *GDF15* levels with age [30]. This connection highlights its role in propagating systemic inflammation and tissue dysfunction during aging. Plasma proteomics studies have validated *GDF15* as a reliable circulating biomarker of aging [25, 30, 31]. Its elevation across diverse aging cohorts underscores its systemic relevance. In clinical biomarker studies, *GDF15* has been consistently identified as a predictor of age-related conditions. Elevated *GDF15* levels are associated with frailty [31–33], sarcopenia [34–36], cardiovascular disease [37–43], and metabolic disorders [44–47]. Importantly, *GDF15* is also linked to all-cause mortality in aging populations, suggesting its potential as a marker of biological age [48]. In particular it has been linked to cardiovascular aging: *GDF15* is strongly associated with cardiovascular outcomes, including heart failure, myocardial infarction, and atherosclerosis. In these contexts, elevated levels reflect heightened inflammatory and fibrotic activity, as well as metabolic stress, particularly in the myocardium and vascular tissues. Elevated *GDF15* levels during aging are associated with metabolic dysregulation, including impaired lipid metabolism and insulin resistance. These findings link *GDF15* to key pathways underlying metabolic aging. In the central nervous system, *GDF15* plays a multifaceted role in neuroinflammation and neurodegeneration [49]. Elevated levels of *GDF15* have been consistently observed in patients with neurodegenerative and cerebrovascular conditions such as Alzheimer's disease (AD), Parkinson's disease (PD), and various forms of dementia [49–53]. These elevated plasma concentrations correlate strongly with disease progression and cognitive decline. Large-scale studies, including the Whitehall II study, the Atherosclerosis Risk in Communities (ARIC) study, and the UK Biobank, have further substantiated the link between increased GDF15 levels and an elevated risk of developing dementia, highlighting its potential as a biomarker for neurodegenerative disease risk and progression [54–58]. Beyond its role as a biomarker, *GDF15* presents opportunities for therapeutic intervention. Inhibiting *GDF15* activity has been proposed as a strategy to mitigate mitochondrial and metabolic stress, potentially reversing or slowing aging-related decline. However, as *GDF15* also may limit acute inflammation in a context-dependent manner [59–65], therapeutic approaches must carefully balance its regulation to avoid unintended consequences.

### EDA2R and cardiometabolic aging

Alongside *GDF15*, *EDA2R* (Ectodysplasin A2 receptor) emerged as a critical marker of cardiometabolic aging, highlighting its potential as both a diagnostic biomarker and a therapeutic target. *EDA2R*, a member of the TNF receptor family, plays a significant role in inflammatory and metabolic pathways that are pivotal during aging [66]. Ectodysplasin A (EDA), a member of the TNF ligand family, plays a key role in the development of ectodermal derivatives during prenatal growth [66, 67]. It exists in two splice variants: EDA-A1, which activates the NF-kB signaling pathway via the EDAR receptor and its adapter protein EDARADD, and EDA-A2, which binds to the *EDA2R* (X-linked ectodermal dysplasia receptor, XEDAR). Beyond its developmental role, EDA-EDA2R signaling has been intricately linked to conditions such as obesity, insulin resistance, and muscle atrophy [66–70], which collectively contribute to cardiometabolic health decline in aging populations. Transcriptomic analyses from cardiometabolic tissues, including heart, liver, skeletal muscle, and adipose tissue, have underscored *EDA2R*'s consistent upregulation across these systems. Notably, its involvement in inflammatory signaling pathways [71] aligns with age-associated increases in systemic inflammation (inflammaging). *EDA2R*'s role extends beyond isolated tissues; data from external datasets reveal significant correlations between EDA2R expression and clinical cardiometabolic metrics such as lipid profiles, cardiovascular markers, and body composition indices [70, 72]. *EDA2R*'s systemic relevance is reinforced by its association with critical age-related health markers and age-related diseases of other organs as well [72–74]. For example, a recent study identified *EDA2R* as a potential biomarker for dementia among participants from the English Longitudinal Study of Ageing (ELSA) through a population-based proteomics approach [73]. Similar results were also reported by other investigative teams [74]. The identification of *EDA2R* as a biomarker has profound implications for understanding the molecular drivers of aging and developing targeted interventions. Its consistent expression across tissues [75] and strong correlation with clinical metrics makes *EDA2R* a promising candidate for multi-system diagnostic panels. Targeting *EDA2R*-mediated pathways could mitigate inflammation and metabolic decline, addressing core aspects of cardiometabolic aging.

### Strengths, limitations, and future directions

This study highlights the power of large-scale transcriptomics to uncover systemic and tissue-specific drivers of aging. The identification of *GDF15* and *EDA2R* as robust biomarkers underscores their utility in diagnostics and their potential as therapeutic targets. *GDF15* stands out as a whole-body biomarker, while *EDA2R* provides specific insights into cardiometabolic aging. These findings pave the way for further studies to validate their roles in aging biology and explore their therapeutic applications.

The integration of transcriptomics with network and machine-learning analyses represents a major strength of this study, enabling a comprehensive view of aging across tissues. The use of GTEx data ensures high-resolution insights into tissue-specific processes, while the focus on cardiometabolic tissues addresses a key area of aging-related disease burden. However, limitations include the cross-sectional nature of the dataset, which precludes longitudinal tracking of gene expression changes. Additionally, while external validation through the SCAPIS-SciLifeLab and NIH AllofUs datasets strengthens the findings, experimental studies are needed to establish causal roles for *GDF15* and *EDA2R* in aging.

Future research should prioritize longitudinal studies to capture dynamic changes in gene expression over time. Multi-omics approaches integrating proteomics, metabolomics, and epigenomics will also provide a more holistic understanding of aging mechanisms. Further validation of *GDF15* and *EDA2R* in diverse populations and experimental models will be critical to their translation into clinical applications.

## Conclusion

This study provides a comprehensive framework for understanding the molecular drivers of aging across tissues. The identification of systemic and tissue-specific markers, including *GDF15* and *EDA2R*, highlights their potential as diagnostic tools [76] and therapeutic targets. By linking transcriptomic insights to clinical outcomes, this work lays the groundwork for future interventions aimed at promoting healthy aging and mitigating the burden of age-related diseases.

## Supplementary Information

Below is the link to the electronic supplementary material.

Supplementary file1 (EPS 1560 KB)

Supplementary file2 (EPS 340 KB)

Supplementary file3 (XLSX 1290 KB)

Supplementary file4 (XLSX 32670 KB)
